# Supplementary material for: High-Resolution Microstructure Characterization of Additively Manufactured X5CrNiCuNb17-4 Maraging Steel during Ex and In Situ Thermal Treatment
Source: Materials (Basel). 2021 Dec 16;14(24):7784. doi: 10.3390/ma14247784 (PMC8707314; doi:10.3390/ma14247784)
Supplement: Supplementary file 1 [file materials-14-07784-s001.zip › materials-1489563-supplementary.pdf]

coatings

Supplementary Materials: High-Resolution Microstructure Characterization of Additively Manufactured X5CrNiCuNb17-4 Maraging Steel during Ex and In Situ Thermal Treatment

Mihaela Albu [1,*], Bernd Panzirsch [2], Hartmuth Schröttner [3], Stefan Mitsche [3], Klaus Reichmann [4], Maria Cecilia Poletti [5] and Gerald Kothleitner [1,3]

[1] Graz Centre for Electron Microscopy, Steyrergasse 17, 8010 Graz, Austria; gerald.kothleitner@felmi-zfe.at
[2] ÖGI- Austrian Foundry Institute, Parkstraße 21, 8700 Leoben, Austria; bernd.panzirsch@ogi.at
[3] Institute of Electron Microscopy and Nanoanalysis, TU Graz, Steyrergasse 17, 8010 Graz, Austria; hartmuth.schroettner@felmi-zfe.at (H.S.); stefan.mitsche@felmi-zfe.at (S.M.)
[4] Institute for Chemistry and Technology of Materials, TU Graz, Stremayrgasse 9, 8010 Graz, Austria; k.reichmann@tugraz.at
[5] Institute of Materials Science, Joining and Forming, TU Graz, Kopernikusgasse 24, 8010 Graz, Austria; cecilia.poletti@tugraz.at
* Correspondence: mihaela.albu@felmi-zfe.at; Tel.: +43-316-873-8348

Citation: Albu, M.; Panzirsch, B.; Schröttner, H.; Mitsche, S.; Reichmann, K.; Poletti, M.C.; Kothleitner, G. High-Resolution Microstructure Characterization of Additively Manufactured X5CrNiCuNb17-4 Maraging Steel during *Ex* and In Situ Thermal Treatment. *Materials* **2021**, *14*, 7784. https://doi.org/10.3390/ma14247784

Academic Editor: Damon Kent

Received: 16 November 2021
Accepted: 13 December 2021
Published: 16 December 2021

Publisher's Note: MDPI stays neutral with regard to jurisdictional claims in published maps and institutional affiliations.

Copyright: © 2021 by the authors. Licensee MDPI, Basel, Switzerland. This article is an open access article distributed under the terms and conditions of the Creative Commons Attribution (CC BY) license (http://creativecommons.org/licenses/by/4.0/).

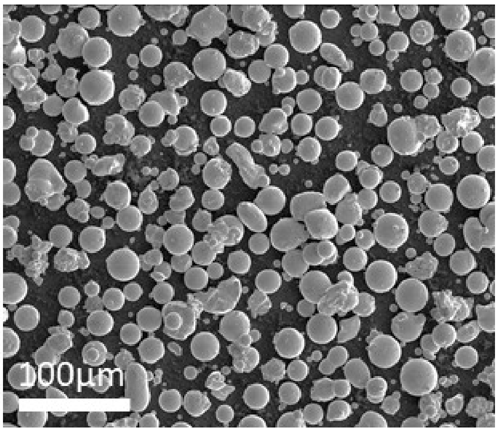

Figure S1. Overview SEM image of the as-received powder.

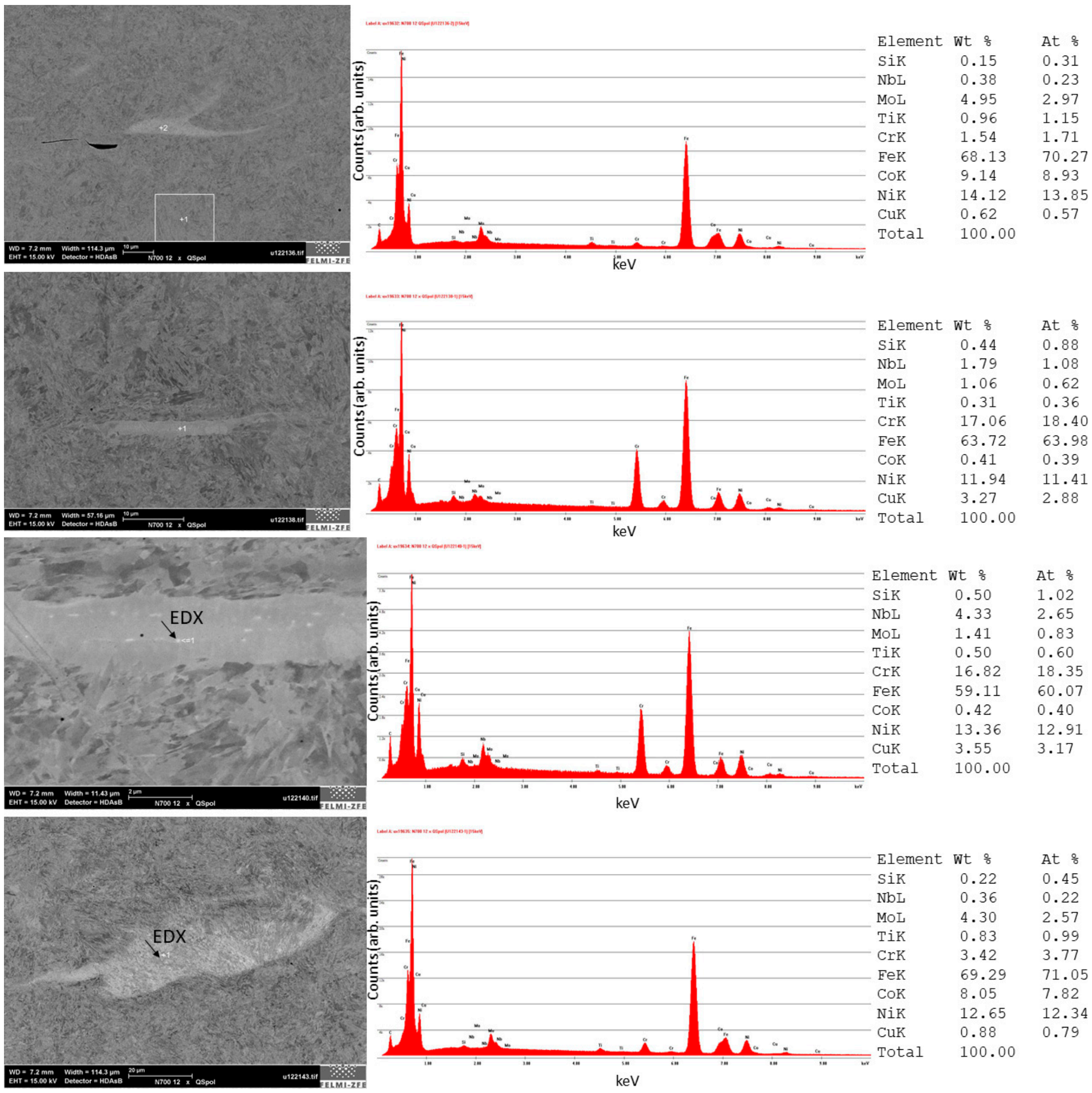

Element	Wt %	At %
SiK	0.15	0.31
NbL	0.38	0.23
MoL	4.95	2.97
TiK	0.96	1.15
CrK	1.54	1.71
FeK	68.13	70.27
CoK	9.14	8.93
NiK	14.12	13.85
CuK	0.62	0.57
Total	100.00	

Element	Wt %	At %
SiK	0.44	0.88
NbL	1.79	1.08
MoL	1.06	0.62
TiK	0.31	0.36
CrK	17.06	18.40
FeK	63.72	63.98
CoK	0.41	0.39
NiK	11.94	11.41
CuK	3.27	2.88
Total	100.00	

Element	Wt %	At %
SiK	0.50	1.02
NbL	4.33	2.65
MoL	1.41	0.83
TiK	0.50	0.60
CrK	16.82	18.35
FeK	59.11	60.07
CoK	0.42	0.40
NiK	13.36	12.91
CuK	3.55	3.17
Total	100.00	

Element	Wt %	At %
SiK	0.22	0.45
NbL	0.36	0.22
MoL	4.30	2.57
TiK	0.83	0.99
CrK	3.42	3.77
FeK	69.29	71.05
CoK	8.05	7.82
NiK	12.65	12.34
CuK	0.88	0.79
Total	100.00	

Figure S2. SEM images and EDX analysis of segregations at the melt pool interfaces and from a NbC particle found inside such a segregation for the as-built sample.

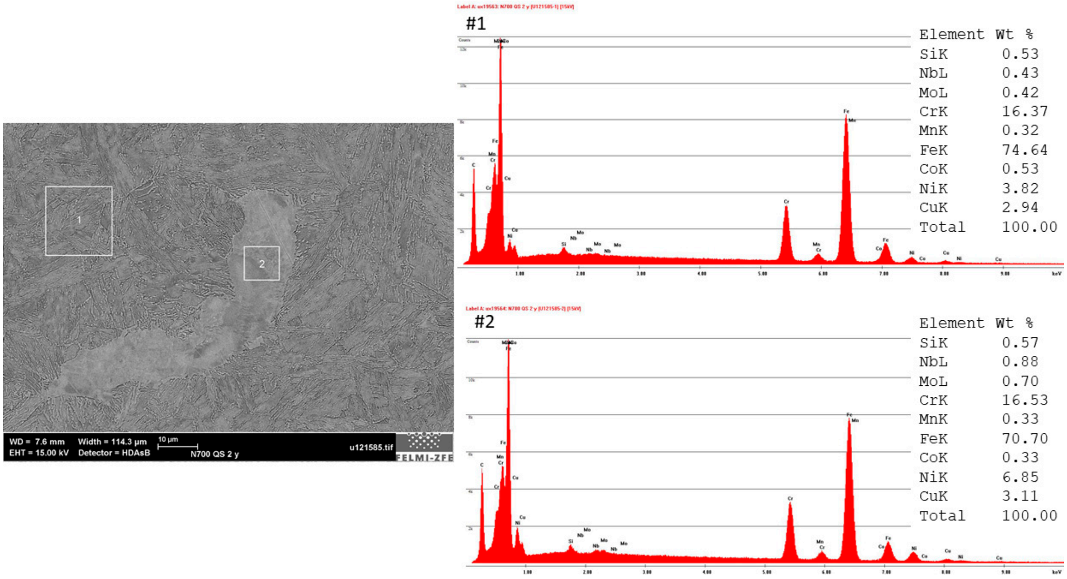

Element	Wt %
SiK	0.53
NbL	0.43
MoL	0.42
CrK	16.37
MnK	0.32
FeK	74.64
CoK	0.53
NiK	3.82
CuK	2.94
Total	100.00

Element	Wt %
SiK	0.57
NbL	0.88
MoL	0.70
CrK	16.53
MnK	0.33
FeK	70.70
CoK	0.33
NiK	6.85
CuK	3.11
Total	100.00

Figure S3. SEM images and EDX analysis of the matrix and segregations for the thermally treated sample.

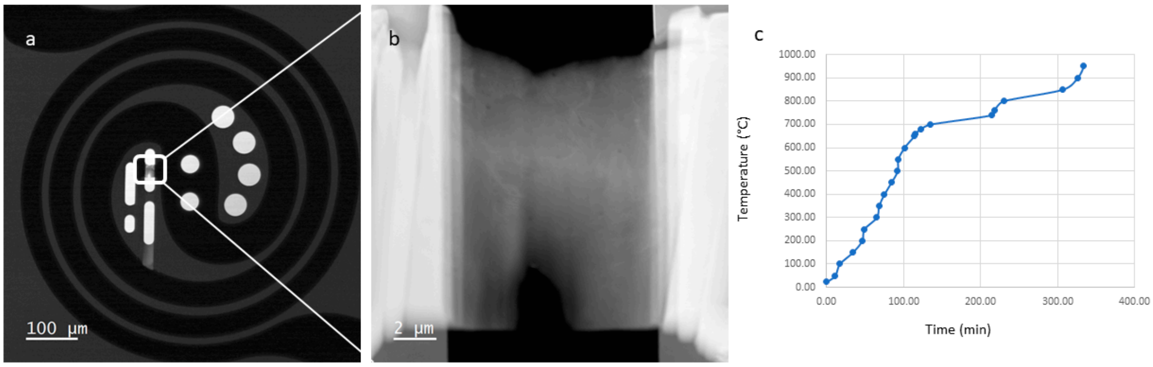

Figure S4. (**a**) heating chip with the FIB Lamella; (**b**) FIB lamella with thick edges; (**c**) heating ramp for *in situ* STEM.

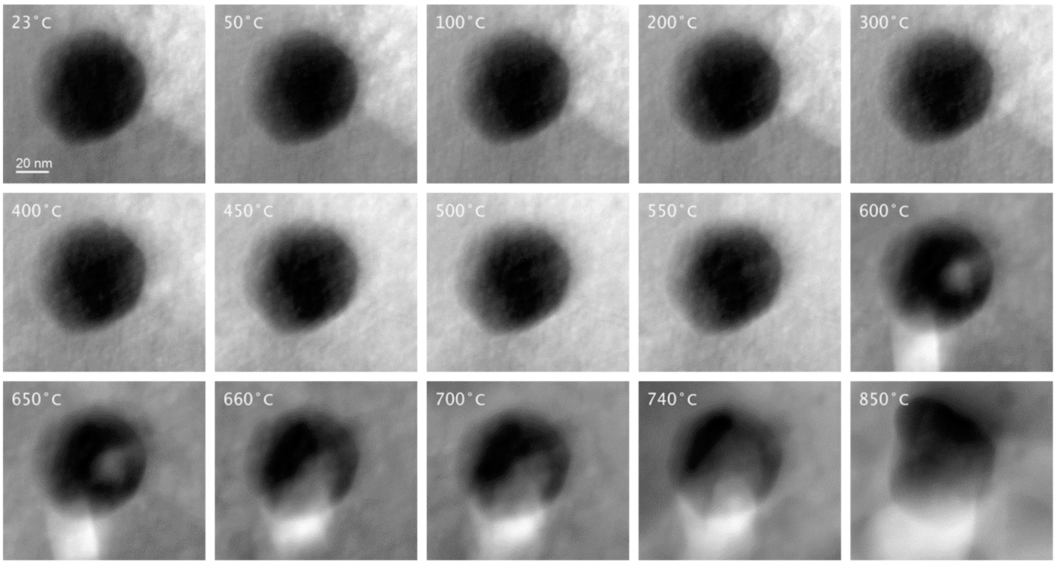

Figure S5. High resolution HAADF images of an oxide nanoparticle during *in situ* heating in STEM.
